# Association of Diarrheagenic *Escherichia coli* Virulence Genes and Antimicrobial Resistance Genes in an Interface Model of Swine Colonization and Human Diarrhea in Mexico

**DOI:** 10.3390/microorganisms13112436

**Published:** 2025-10-23

**Authors:** Elsa M. Tamayo-Legorreta, Eduardo Moreno-Vazquez, Jackeline Cerón-López, Fabian Tellez-Figueroa, Rosa M. Medina-Julián, Hugo López-Gatell, Celia M. Alpuche-Aranda

**Affiliations:** 1 Centro de Investigación Sobre Enfermedades Infecciosas, Instituto Nacional de Salud Pública, Cuernavaca 62100, Morelos, Mexico; emtamayo@insp.mx (E.M.T.-L.); emorenov9@gmail.com (E.M.-V.); jackeline-2013@hotmail.com (J.C.-L.); luis.tellez@insp.mx (F.T.-F.); hugo.lopez-gatell@insp.mx (H.L.-G.); 2 Laboratorio Estatal de Salud Pública, Servicios de Salud Morelos, Jiutepec 62573, Morelos, Mexico; ubaldosoto52@hotmail.com

**Keywords:** *Escherichia coli*, antimicrobial resistance, pathotypes, swine, human

## Abstract

The accelerated spread of antimicrobial resistance, driven by the misuse of antibiotics in the context of ‘One Health’, is a public health concern worldwide due to the increasing number of human infections associated with foodborne and/or environmental pathogens, including multidrug-resistant *Escherichia coli* (*E. coli*). Monitoring pathogenic and multidrug-resistant *E. coli* isolates is essential for sustainable disease management in swine and human diarrhea cases. This study was designed to assess the multidrug resistance (MDR) profiles and virulence-associated gene (pathotypes) frequency of pathogenic and commensal *E. coli* strains by antimicrobial susceptibility testing and endpoint PCR among 983 *E. coli* isolates from swine fecal material and 425 stool isolates from human diarrhea cases, obtained from a closely monitored population between March 2015 and April 2016. Our results reveal that >50% of *E. coli* strains isolated from swine were resistant to nalidixic acid (78.94%), tetracycline (72%), ampicillin (55.54%), and co-trimoxazole (53.91%), and that, in humans, the highest resistance was observed in tetracycline (71.77%), nalidixic acid (65.41%), ampicillin (57.88%), and co-trimoxazole (53.88%). A lower frequency of resistance to ciprofloxacin was demonstrated in both swine (23.4%) and humans (15.3%), and minimal resistance to third-generation cephalosporins, ceftazidime (2.54%), and cefotaxime (2.44%) was observed in swine; however, resistance to these cephalosporins is much higher at 14.6% and 11.53% in humans. Among the pathotypes, EPEC was the most predominant (70.97%) in swine and DAEC (40%) in humans. In addition, pulsed-field gel electrophoresis separates the *E. coli* isolates into 22 patterns. Pathotypes such as EPEC and EHEC in swine highlight the need for surveillance and control in animal production to prevent zoonotic transmission. These results suggest that swine could act as a reservoir in human infection and that antimicrobial resistance could be transferred to humans from swine. Although we did not find clonal dissemination between the human and swine strains, the spread of similar multi-resistance profiles was observed, thus suggesting that multidrug resistance has been widely selected in this closed environment and may pose a public health risk.

## 1. Introduction

The constant evolution of infection-causing microorganisms, adapting to new hosts and environments, and crossing the barrier of specificity from colonization or infection in animals to humans or vice versa, imposes a new and additional toll of disease on health systems. These threats to human and animal health are triggered by multiple, interrelated, global factors related to human behavior, environmental changes, and the characteristics of the genetic plasticity of microorganisms. These factors reflect the complexity of ecosystems in which humans and animals share space. Understanding this complex interaction to develop and implement prevention and control measures against these infections cannot be tackled from a single angle. Therefore, the multidisciplinary approach converging in the “One Health” concept is essential. This concept recognizes the intrinsic link between human, animal, and environmental health and emphasizes a collaborative, multisectoral approach to addressing global health challenges. One Health provides a comprehensive framework for understanding and mitigating antimicrobial resistance, zoonotic diseases, and other health threats that arise at the human–animal–environment interface [1,2,3].

We have broadly discussed the selection of antimicrobial-resistant bacteria in animals and humans using various antibiotics as a clinical treatment in humans or animals or their use in animals as growth promoters. In addition, it has been suggested that the widespread use of antibiotics as growth promoters in animals exposes the human population to subtherapeutic doses through the consumption of animal products or by disseminating selected resistant strains in animals to various sources in the environment [4,5]. An example of bacteria with increasing antibiotic resistance is Enterobacteriaceae, such as *Escherichia coli*, one of the critical threats to global public health, generating significant morbidity and mortality and associated prolonged healthcare costs [6,7]. *E. coli* is a ubiquitous bacterium in the intestinal tract of humans and warm-blooded animals [8,9]. *E. coli* strains associated with widely community-acquired infections, such as acute enteritis, have been classified based on the presence of virulence-associated genes (VAGs) into six different pathogenic groups: Enteropathogenic *E. coli* (EPEC), enterohemorrhagic *E. coli* (EHEC), enterotoxigenic *E. coli* (ETEC), enteroinvasive *E. coli* (EIEC), enteroaggregative *E. coli* (EAEC), and diffusely adherent *E. coli* (DAEC) [10,11,12,13,14]. 

The emergence and persistence of multidrug-resistant (MDR) *E. coli* causing acute diarrhea is a serious public health problem. It causes nearly 500,000 deaths per year, especially among children up to five years of age in Asia, Africa, and Latin America [11,15]. The incidence differs between countries and regions due to recognized factors, such as socioeconomic group, sewage disposal, food safety, and close contact with animal reservoirs of potential pathogens [11,16,17,18,19].

The selection of antibiotic-resistant-pathogenic *E. coli* has increased due to horizontal gene transfer and exchange of mobile genetic elements, which has had significant public health implications [20]. These healthcare-acquired MDR bacteria have spread to livestock and the environment due to the persistence of antibiotic-resistance genes found in mobile genetic elements (MGEs) capable of spreading efficiently among bacteria in the community environment [21].

Identifying MDR or highly resistant bacteria (XDR) in pig farms alongside related communities could be considered as a monitoring system for the early detection of this event. Furthermore, the presence and persistence of these bacteria in swine could describe an important potential vehicle for developing and spreading antimicrobial resistance, especially in environments with inadequate sanitation [22].

This study aimed to assess the multidrug resistance (MDR) profiles and virulence-associated gene (pathotypes) frequency of pathogenic and commensal *E. coli* strains in the swine population of a semi-efficient farm and the simultaneous intensified epidemiological surveillance of acute diarrhea in the people living adjacent to the farm and consuming its products located in Jiutepec, Morelos, Mexico. This scientific evidence is needed to support the more specific regulation of antimicrobial use and surveillance of diarrheal disease in an interface model of swine colonization and human diarrhea in Mexico or similar countries.

## 2. Materials and Methods

### 2.1. Study

A descriptive cross-sectional study was carried out in Jiutepec, Morelos, Mexico. We included a semi-efficient swine farm (where swine production and consumption occur in the same community) and a model of epidemiological surveillance of diarrheal disease in the population attending three primary healthcare centers (PHC) located near the swine farm: Jiutepec, Huizachera, and Calera Chica. Moreover, the Cuernavaca General Hospital (HGC) “Dr. José G. Parres” was included, which referred patients with moderate and severe diarrhea cases from the region surveyed who did not attend the HCs.

### 2.2. Sample

Fecal samples of all pigs on the farm were obtained twice with an interval of six months. The first sampling was conducted from March to April 2015, and we collected 280 porcine fecal samples, and the second collection period took place in September 2015 with 228 fecal samples. For each pig, one rectal swab was obtained, and, using Cary–Blair transport medium (Deltalab, Barcelona, ES), the swabs were transported in cold boxes to the Laboratory of Infectious Disease Epidemiology of the National Institute of Public Health. Samples were inoculated on McConkey agar culture medium within four hours of collection. In addition, we collected 262 fecal samples from patients attending any of the three HCs or the CGH “Dr. José G. Parres”. The surveillance period was from May 2015 to April 2016. We included people with acute diarrheal disease who sought medical care from Monday through Friday from 8:00 to 16:00 at the three HCs and the General Hospital of Cuernavaca. They received routine medical care at these HCs, and we asked each of them for consent to participate in the study. From each participant, a rectal swab was taken, and, using Cary–Blair medium (Deltalab, Barcelona, ES), the swabs were transported in cold boxes (4 °C) to the State Public Health Laboratory for processing within four hours of arrival. The Research, Ethics, and Biosafety Committees approved this study at the National Institute of Public Health with CI: 1270, CE: 1642, and CB 1261, respectively.

### 2.3. Isolation and Identification of Escherichia coli Strains

Fecal samples were inoculated on selective MacConkey agar medium and cultured overnight at 37 °C (Thermo Scientific, Iowa City, IA, USA) to isolate *E. coli*. Three separate well-defined colonies with *E. coli* appearance (lactose positive and colony morphology) were randomly selected and re-inoculated on MacConkey agar and cultured at 37 °C overnight. API 20E (Biomérieux, Salt Lake City, UT, USA) was used to identify *E. coli*, incubated at 35 °C for 18 h and identified according to the manufacturer’s reading and interpretation criteria.

### 2.4. Antimicrobial Susceptibility Testing

Microdilution and agar disk diffusion methods were used to perform susceptibility testing on 10 antimicrobial agents according to Clinical and Laboratory Standards Institute (CLSI) guidelines [23]. The antimicrobial agents used included ampicillin (AMP; 10 µg), ceftazidime (CAZ; 30 µg), cefotaxime (CTX; 30 µg), imipenem (IPM; 10 µg), amikacin (AMK; 30 µg), gentamicin (GEN; 10 µg), ciprofloxacin (CIP; 5 µg), nalidixic acid (NA; 30 µg), trimethoprim/sulfamethoxazole (SXT, co-trimoxazole; 25 µg), and tetracycline (TET; 30 µg). All assays were performed in duplicate. Minimum Inhibitory Concentration (MIC) and disk diffusion results were interpreted according to the breakpoints provided by the CLSI [23] and classified as susceptible, intermediate, or resistant. These antimicrobials are representative of the major antimicrobial drug classes that are important for both veterinary and human medicine. *E. coli* ATCC 25922 and *Klebsiella pneumoniae* ATCC 700603 were used as controls. Classification of MDR strains was defined as resistance to at least three or more antibiotic families [24,25].

### 2.5. Virulence Factor Gene Detection

Virulence genes to determine six different *E. coli* pathotypes were identified by conventional PCR. Bacterial boiling method was used to obtain bacteria DNA [26]. The selected virulence genes and the specific primers are shown in Table 1. Each 25 μL reaction mixture contained a final concentration of 1x reaction buffer (100 mM Tris/HCl, pH 8.5; 500 mM KCl, 1% Triton X-100), 2 mM MgCl_2_, 0.2 mM each dNTP (dNTP mix, 10 mM, Thermo Scientific, Iowa City, IA, USA), 10 μM each primer, 1.0 U Taq DNA polymerase (Thermo Scientific, Iowa City, IA, USA), 5 μL template DNA, and nuclease-free water. The PCR amplification conditions for all reactions were initial denaturation at 94 °C for 5 min, followed by 30 cycles of denaturation at 94 °C for 45 s, annealing at 58 °C for 45 s, and extension at 72 °C for 45 s, and a final extension at 72 °C for 7 min [16]. PCR products were visualized by electrophoresis on a 1% agarose gel stained with SYBR^®^ Green (Invitrogen, Carlsbad, CA, USA) at 100 V for 45 min. The positive controls were EPEC O127:H6 (strain E2348/69, *eae*), EHEC O157:H7 (*eae*, *stx*1, *stx*2), ETEC O78:H11 (strain H10407, *elt*, *est*), EIEC O136:NM (*ipa*H), EAEC O44:H18 (strain O42, *agg*R), and DAEC O75:H-E66438 (*daa*E) (kindly donated by Dr. Carlos Eslava, Universidad Nacional Autónoma de México, Mexico City 04510, Mexico).

### 2.6. Pulsed-Field Gel Electrophoresis (PFGE)

MDR-EC strains were genotyped using PFGE [28,29]. We prepared the genomic DNA with the bacterial boiling method and digested it with 50 U *Xba*I (New England BioLabs Inc., Ipswich, MA, USA). DNA was separated by electrophoresis using a CHEF-DR II system (BioRad, Birmingham, UK). After 23 h, the gel was stained with ethidium bromide, and PFGE patterns were visualized under UV light. We used the *Salmonella* serotype Braenderup strain (H9812) as a control and the Lambda PFG ladder (New England BioLabs Inc., Ipswich, MA, USA) as a size marker. PFGE patterns were interpreted following the criteria of Tenover [30]. In addition, a computer analysis of PFGE profiles was performed with BioNumerics 5.10 software (Applied Maths, Sint-Martens-Latem, Belgium). Dendrograms were constructed using Dice’s similarity coefficient, a tolerance coefficient, and unweighted pair group methods with the arithmetic mean algorithm (UPGMA).

### 2.7. Statistical Analysis

We performed a two-sample proportion test (Z-test for proportions) to compare the antimicrobial resistance rates and *Escherichia coli* virulence gene frequency between farm animals and the human population. This test evaluates whether two independent proportions are significantly different by calculating a Z-score based on the standard normal distribution [31]. A two-sample test for proportions (Z-test for proportions) was used to compare the proportions of antimicrobial resistance between different groups. This statistical method is appropriate when evaluating differences between two independent groups with categorical binary outcomes (e.g., presence or absence of resistance genes). The Z-test for proportions assumes a sufficiently large sample size to approximate the normal distribution under the central limit theorem, making it a robust method for comparing proportions in microbiological and epidemiological studies [31]. The test was performed at a 95% confidence level, and significance was assessed at *p* < 0.05.

## 3. Results

### 3.1. Prevalence of Escherichia coli Strains Isolated from Swine and Human

A total of 1524 presumptive *E. coli* strains were isolated from 508 swine fecal samples, of which 983/1524 (64.5%) were confirmed by API 20E. Similarly, 786 presumptive *E. coli* strains were isolated from 262 human diarrheal cases, of which 425/786 (54.07%) were confirmed by API 20E. Most of the swine *E. coli* strains came from piglets (517/983, 52.59%) and sows (444/983, 45.17%), while human isolates were mainly obtained from patients at the Cuernavaca General Hospital “Dr. José G. Parres” (Table 2).

### 3.2. Antimicrobial Susceptibility Testing

Table 3 shows the antimicrobial susceptibility results for pathogenic (PCR-pathogenic-marker-positive) and non-pathogenic *E. coli*. A comparative resistance analysis revealed a significantly higher resistance in swine isolates to nalidixic acid (78.94% vs. 65.41%; *p* < 0.0001) and ciprofloxacin (23.39% vs. 15.3%; *p* = 0.0007). In contrast, human isolates showed a significantly higher resistance to gentamicin (46.35% vs. 37.84%; *p* = 0.0032), ceftazidime (14.59% vs. 2.54%; *p* < 0.0001), cefotaxime (11.53% vs. 0%; *p* < 0.0001), and ceftazidime/cefotaxime (11.29% vs. 2.44%; *p* < 0.0001) than porcine isolates. No significant differences were observed for ampicillin (*p* = 0.42) and cotrimoxazole (*p* = 0.95), while tetracycline resistance remained equally high in both populations (72%; *p* = 0.98). Multidrug resistance was detected in 66.22% of swine isolates and 64.71% of human isolates, with no significant difference (*p* = 0.61). Resistance to amikacin and imipenem was absent in all porcine and human isolates.

### 3.3. Multidrug Resistance

Table 4 shows the results of the identified multidrug resistance profiles. We observed that 25.54% of the swine isolates were resistant to three antibiotic families (251 isolates). The most common resistance profile was NAL-SXT-TET (48 isolates), followed by GEN-NAL-TET (43 isolates), while 22.35% (95 human isolates) were shown to be resistant to three antibiotic families; the most common resistance profiles were AMP-NAL-TET and GEN-NAL-TET (19 isolates). Resistance to eight antibiotics was found in only six (0.61%) swine and eighteen (4.24%) human isolates. Regarding multidrug resistance, among those resistant to three or more drugs out of the seven tested, 66.22% (651 swine isolates) and 64.71% (275 human isolates) showed multidrug resistance.

### 3.4. Pathotype Characterization

The determination of pathotype-specific genes showed that 6.31% (62/983) and 22.35% (95/425) of *E. coli* isolates from swine and humans, respectively, carried at least one pathogenicity marker (Table 5). The highest percentage of diarrheagenic *E. coli* (DEC) was isolated from humans. The most common pathotypes in swine were EPEC (*eae*), associated with diarrhea, and EHEC (*stx*1, *stx*2, and *stx*1: *stx*2), associated with hemorrhagic colitis. In humans, DAEC (*daa*) was the most frequent pathotype, followed by EPEC (*eae*).

### 3.5. Phylogenetic Analysis of E. coli

**Figure 1:**
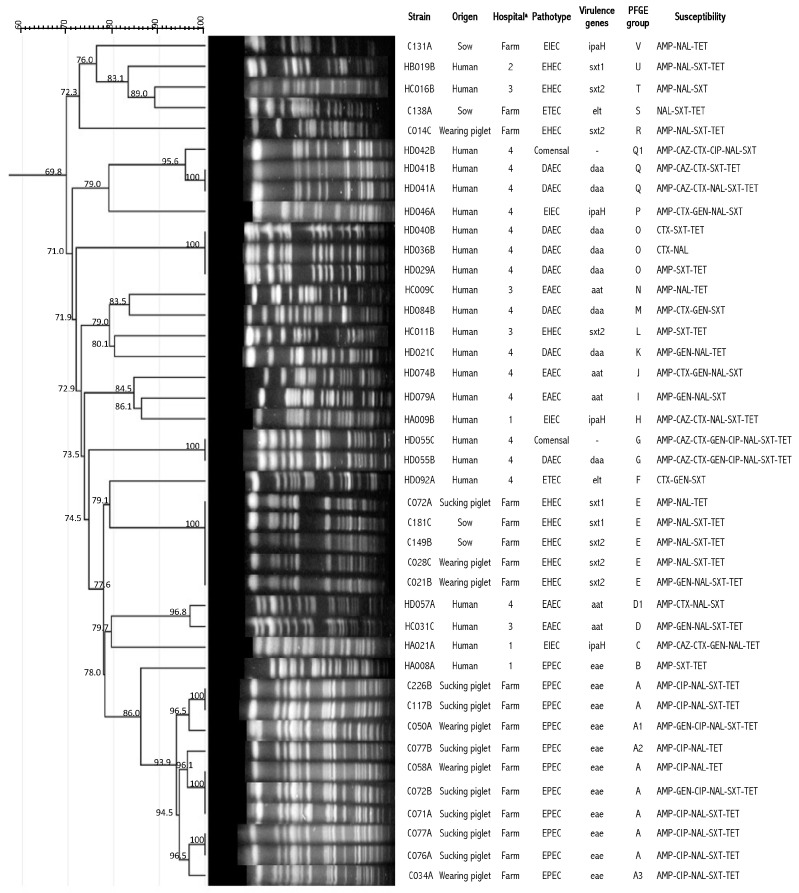
Genetic diversity of *E. coli* strains isolated from swine and human fecal samples in Jiutepec, Morelos, Mexico, from 2015 to 2016. The pathotype pattern of *E. coli* from swine was closely associated with EPEC and EHEC pathotypes, but the PFGE pattern of human *E. coli* was discrete. The dendrogram was elaborated using the Dice coeIicient and the UPGMA (arithmetic mean algorithm). PFGE using *Xba*I endonuclease. ^a^ Health Centres for sample collection in Jiutepec, Morelos, Mexico: 1-CS Jiutepec, 2-CS Huizachera, 3-CS Calera Chica, 4-Hospital General de Cuernavaca ”Dr José G. Parres”, Cuernavaca, Morelos, Mexico.

The PFGE analysis revealed 22 patterns among the *E. coli* isolates from swine and humans (A–V) with five subtypes (A1–A3, D1, and Q1). The patterns (A, A1–A3, E, R, S, and V) were present in *E. coli* MDR isolates from swine, whereas the patterns (B, C, D, D1, F, G, H, I, J, K, L, M, N, O, P, Q, Q1, T, and U) were present in isolates from the community population. The most represented patterns from swine were A (ten isolates) and E (five isolates), while those from humans were O and Q, each with three isolates; D and G, each with two isolates; and the remaining patterns from both swine and humans were represented by only one isolate. We found that most of the patterns (A and E) were present only in piglets and sows. At the same time, the MDR *E. coli* isolates did not show clonality in the human isolates, except for ten isolates (D, D1, G, O, Q, and Q1). The results of the computer analysis of the banding patterns showed similarity percentages ranging from 100% to 70% (Figure 1). When the majority of the PFGE patterns were compared with the antimicrobial susceptibility profile and pathotypes, we observed that the isolates with patterns A and E were sensitive to ceftazidime and cefotaxime and were EPEC and EHEC. Isolates with patterns G, O, and Q were resistant to third-generation cephalosporins. All isolates were sensitive to imipenem and amikacin.

## 4. Discussion

A comparative analysis of antimicrobial resistance between *Escherichia coli* isolates from swine and humans reveals distinct patterns consistent with the findings of recent studies [32,33]. In our research, swine isolates exhibited a significantly higher resistance to nalidixic acid (78.94% vs. 65.41%; *p* < 0.0001) and ciprofloxacin (23.39% vs. 15.3%; *p* = 0.0007) compared to human isolates. Fluoroquinolones are classified as “critically important antimicrobials” due to their significance in human and animal medicine [34,35]. The widespread use of fluoroquinolones and enrofloxacin (nalidixic acid precursor) in veterinary medicine may explain this increased resistance in swine. Similarly, a study correlating antimicrobial resistance patterns of *E. coli* in livestock and humans reported lower resistance rates to nalidixic acid in *E. coli* strains isolated from swine (21.1%) and humans (15.8%) [36]. Ciprofloxacin resistance in humans (15.3%) was low compared to other studies that reported a 32% resistance to ciprofloxacin in *E. coli* isolates from children < 5 years of age from the National Institute of Pediatrics in Mexico City [37]. Our results for the nalidixic acid resistance (78.94%, 65.41%) of *E. coli* isolates from swine and humans, respectively, were higher than those of other countries, e.g., Netherlands: 1.0%, and Korea: 65.6% [33,38]. High quinolone resistance in swine is a critical problem, suggesting an extensive use of antibiotics in animal husbandry. It could drive a high selective pressure towards bacteria resistant to these antibiotics. Since quinolone resistance can be transmitted from swine to humans, this becomes a public health hazard. A possible explanation for the high proportion of bacteria resistant to quinolones and ampicillin, even though these antibiotics are not administered directly to the swine, could be the presence of certain antibiotics (e.g., tetracyclines) in their feed. This may be indirectly selected for resistant bacteria through resistance cassettes, contributing to the persistence and dissemination of antimicrobial resistance within the swine population. Reducing antimicrobial resistance is crucial for preventing the transmission of resistant strains from animals to humans and maintaining treatment efficacy [39].

In contrast, human isolates in our study had a significantly higher resistance to gentamicin (46.35% vs. 37.84%; *p* = 0.0032), ceftazidime (14.59% vs. 2.54%; *p* < 0.0001), and cefotaxime (11.53% vs. 0%; *p* < 0.0001) than porcine isolates. This trend is consistent with the results of another study in which higher levels of resistance to most antimicrobial agents were found in humans than in swine isolates [40]. In contrast, data published in Korea showed that *E. coli* strains isolated from swine showed a 51.6% resistance to gentamicin, whereas those isolated from human patients showed a lower resistance [33,41]. A U.S. study also observed a high gentamicin resistance in porcine *E. coli* strains [9]. The contrast with the current published data could indicate geographic variations or differences in antibiotic use among the populations studied.

One of the resistances that stood out in our study was that of third-generation cephalosporins (3GC), particularly ceftazidime (CAZ) and cefotaxime (CTX) in swine; this highlights an emerging problem in this farm animal model. The frequency of CAZ and CTX resistance in swine is like that in Thailand [42], and the resistance observed in humans was lower than that reported in other studies [37]. Recent studies have indicated that resistance to 3GC, especially those mediated by extended-spectrum β-lactamases (ESBL), is a worldwide concern in humans and production animals [43,44]. Previous studies suggest that resistance to β-lactams in animals intended for human consumption may indicate their role as a reservoir of resistance genes within mobile genetic elements. The transfer of resistant bacteria to humans is possible through direct contact with animals or the food chain, as suggested by these studies [45]. Thus, it is unlikely that the selection for resistance occurred on the farm, which corroborates the multiclonality results, as selection in a closed environment such as a farm would favor clonal spread. The individuals sampled did not come from hospitalized patients but from community-acquired infections. These data suggest that 3GC resistance is spreading outside the human environment into the swine farm. The prevalence of resistance to 3GC in swine isolates suggests that human antimicrobial use may factor in the emergence of resistant bacteria in animals.

No significant differences were observed in our study for ampicillin (*p* = 0.42) and co-trimoxazole (*p* = 0.99), while resistance to tetracycline remained equally high in both populations (72%; *p* = 0.98). These results are, in part, consistent with those of Christodoulou (2023) [36], who also reported a high tetracycline resistance in *E. coli* isolates from both swine (84.2%) and humans (73.7%). However, contrary to our results, he observed a significant difference in ampicillin resistance between swine (31.6%) and humans (52.6%). The ampicillin resistance in our study suggests a pronounced and non-random issue with this antibiotic in the swine population. According to published studies, the frequency of ampicillin resistance reported in strains from animals (swine) is highly variable, with values lower or higher than those found in our study (55.44%) [46,47,48,49]. In strains isolated from patients with diarrhea, the frequency of resistance found (57.88%) was higher than in South Korea (40%) [50] and lower compared to a study conducted in Mexico, where ampicillin resistance was 69% [37]. This suggests that resistance to this antibiotic is spreading in humans, in strains colonizing animals, and, most likely, in the ecosystem in this model human–animal health interface. Although this study does not allow us to determine the direction of transfer of the resistance mechanism, they likely came from human strains, since ampicillin has long been in use. Transmission may have occurred through water or soil [51,52].

Among *E. coli* isolates recovered from swine and humans, a high resistance to tetracycline and co-trimoxazole (trimethoprim/sulfamethoxazole) was observed, but without statistical significance in either group (*p* > 95). This could indicate widespread resistance. We found a similar tetracycline resistance of 72% in swine and humans, which is high compared to other studies [50]. A comparison of our results in the case of co-trimoxazole resistance indicates a similar prevalence in *E. coli* strains isolated from swine and humans. A recent report by the European Food Safety Authority (EFSA) [53] indicated a high prevalence of co-trimoxazole resistance in *E. coli* strains of animal and human origin, reflecting a trend like the data provided. Some studies showed that co-trimoxazole resistance in *E. coli* of human origin may vary, but remains significant, e.g., in Mexico City (44%) [37] and South Korea (35%) [50].

Our study detected multidrug resistance (MDR) in 66.22% of swine isolates and 64.71% of human isolates, with no significant difference (*p* = 0.61). This high prevalence of MDR is concerning and underscores the need for the prudent use of antimicrobials in human and veterinary medicine. These results were higher than those obtained in other studies in swine [54] and humans [32,55]. In a longitudinal study, significant differences in the prevalence levels of multidrug-resistant isolates were also observed between host species, with swine being at a higher risk of carrying MDR *E. coli* [56]. Our results could reflect a more intensive use of antibiotics in the livestock industry. In addition, a high resistance to combinations of more than three antibiotics is more prevalent in swine, which may indicate a higher selective antibiotic pressure in these animals and more extensive or inadequate exposure to multiple antibiotics in swine. Resistance to more than five antibiotics highlights a serious multi-resistance problem in the animal population and is of concern due to the risk of transmission to humans. The interface between human and animal health is critical in the context of antimicrobial multidrug resistance. This study shows a high prevalence of MDR *E. coli* in swine, which may have significant implications for human health due to the possibility of transferring resistant bacteria or resistance genes [33,57]. The high level of antimicrobial multidrug resistance is directly related to the challenges in the treatment of infectious diseases, making it essential to control it. On the other hand, we did not observe a relationship between the multidrug resistance profile and the presence or absence of genes encoding enteropathogenic factors among *E. coli* isolated from swine and humans. Therefore, the factors that select MDR *E. coli* strains are independent of the pathogenicity of the microorganism.

A gene-specific pathotyping analysis revealed that 6.31% of *E. coli* isolates of swine-origin and 22% of those of human origin carried at least one pathogenicity marker. The prevalence of pathogenicity markers is significantly higher in humans than in swine, which may be attributed to the expected higher frequency of pathogenic *E. coli* in the human cases of diarrhea studied. The most common pathotypes in swine were EPEC (*eae*), associated with diarrhea, and EHEC (*stx*1, *stx*2, and *stx*1:*stx*2), associated with hemorrhagic colitis, suggesting a significant risk to animal health and potentially to human health if zoonotic transmission occurs. Although EPEC was most prevalent in swine, ETEC has been one of the most frequently isolated pathotypes in swine [58]. ETEC is one of the main causes of diarrhea in suckling and weaned piglets, causing essential losses in the swine industry [58,59]. The low prevalence of ETEC found in this study could be because there was no incidence of sick swine with diarrhea. A curious finding was the detection of EIEC on the farm. The detection of EIEC on swine farms could imply a possible transmission from a human source or a change in the dynamics of the pathogen in the farm environment. This issue raises concerns about the possibility of reverse zoonosis, where pathogens are transmitted from humans to animals and vice versa. Such transmission may increase the risk of disease outbreaks in animal and human populations.

In addition, the presence of EIEC in swine could indicate an adaptation of this pathogen to a new host, an example of bacterial evolution. It could suggest that EIEC is developing the ability to survive and multiply in different environments and hosts. EIEC infections can cause economic losses due to decreased animal growth, increased mortality, and costs associated with the treatment and management of outbreaks [60,61]. No swine EAEC (*aat*) and DAEC (*daa*) isolates were found. The most common pathotype in humans was DAEC (*daa*), followed by EPEC (*eae*). Both pathotypes are associated with diarrhea in humans, indicating a significant burden of gastrointestinal disease caused by pathogenic *E. coli*. The difference between the pathotypes suggests variability in the distribution of pathotypes between swine and human populations.

Molecular typing by PFGE investigates the clonal relationship between different bacterial isolates. We identified twenty-two distinct patterns in the *E. coli* isolates, and, despite the diversity, six patterns predominated; this heterogeneity of patterns and predominance of specific clones has been observed in other studies [62,63,64]. The diversity of *E. coli* patterns in swine and humans and the MDR strains in both groups suggest a potential for zoonotic transmission. Swine may act as reservoirs for resistant strains transmitted to humans, especially in close-contact environments such as farms and slaughterhouses. We detected clonal spread on the swine farm, where the *E. coli* strains with the most PFGE patterns were found in suckling piglets, weaning piglets, and sows due to the proximity of swine in the pens.

## 5. Conclusions

The data suggest that MDR has been widely targeted in this closed environment. In swine, dangerous pathotypes such as EPEC, EHEC, and EIEC underline the need for surveillance and control in animal production to prevent zoonotic transmission. Our study on the association of virulence genes and antimicrobial resistance in diarrheagenic and commensal *E. coli* in a swine colonization human diarrhea interface model in Mexico is essential in order to address the complex transmission dynamics of multidrug-resistant bacteria, highlighting the importance of genetic surveillance and the implementation of responsible antibiotic use and stewardship practices to reduce the risk of zoonotic transmission and protect public health. A “One Health” approach is vital in order to address the antimicrobial resistance, pathogenicity, and clonality of *E. coli*. The present study has limitations. The results analyze the sharing of patterns of antibiotic resistance but do not establish the direction of the transfer of this resistance or the mechanisms of transmission between animals and humans. Future research will focus on the whole-genome sequencing (WGS) of multidrug-resistant pathogenic and commensal *E. coli* strains to determine whether different resistance mechanisms are co-selected in this interface model. These efforts will further elucidate the evolutionary pathways of resistance transmission and provide valuable insights in order to improve antibiotic stewardship in human and animal health.

## Figures and Tables

**Table 1:** Primers used in conventional PCR for virulence gene amplification were used to identify six different *E. coli* pathotypes isolated from swine and humans in Jiutepec, Morelos, Mexico, from 2015 to 2016.

Pathotype ^1^	Target Gene	Primer Sequence (5′-3′)	Amplicon Size (bp)	Reference
EPEC	*eae*	F: ACTGGACTTCTTATTTCCGTTCTATG R: CCTAAACGGGTATAATCACCAGA	189	[27]
EHEC	*stx-1*	F: AGTCGTACGGGGATGCAGATAAAT R: CCGGACACATAGAAGGAAACTCAT	418	[27]
	*stx-2*	F: GGCACTGTCTGAAACTGCCC R: TCGCCAGTTATCTGACATTCTG	255	[27]
ETEC	*elt*	F: GGCGACAGATTATACCGTGC R: CGGTCTCTATATTCCCTGTT	440	[27]
	*est*	F: ATTTTTMTTTCTGTATTRTCTT R: CACCCGGTACARGCAGGATT	191	[27]
EIEC	*ipaH*	F: GTTCCTTGACCGCCTTTCCGATACCGTC R: GCCGGTCAGCCACCCTCTGAGAGTAC	619	[27]
EAEC	*aat*	F: AGGTTTGATAATGATGTCCTTGAGGA R: TCAGCTAATAATGTATAGAAATCCGCTGTT	152	[27]
DAEC	*daa*	F: ATTACGTCATCCGGGAAGCACACA R: GCTTGCTCATAAAGCCGCAGACAA	146	[27]

^1^ Abbreviations: EPEC, enteropathogenic *E. coli*; EHEC, enterohaemorrhagic *E. coli*; ETEC, enterotoxigenic *E. coli*; EIEC, enteroinvasive *E. coli*; EAEC, enteroaggregative *E. coli*; DAEC, diffusely adhering *E. coli*.

**Table 2:** Sample sources of collections and *Escherichia coli* isolates detected in swine and humans from in Jiutepec, Morelos, Mexico, from 2015 to 2016.

Source (Number of Samples)	Age Group and/or Healthcare Centers ^1^	Total Number of Fecal Samples	Total Number of Confirmed *E. coli* (API 20E) (%)
Swine Farm (N = 508)	Suckling piglets	160	289 (29.4)
	Weaning piglets	126	228 (23.19)
	Sows	211	444 (45.17)
	Boar	11	22 (2.24)
Human (N = 262)	HC Jiutepec	36	58 (13.65)
	HC Huizachera	47	83 (19.53)
	HC Calera Chica	68	110 (25.88)
	CGH “Dr. José G. Parres”	111	174 (40.94)

^1^ Abbreviations: HC = Health Centers; CGH = Cuernavaca General Hospital “Dr. José G. Parres”.

**Table 3:** Antimicrobials resistance and statistical analysis of diarrheagenic and commensal *E. coli* strains from swine and human in Jiutepec, Morelos, Mexico, from 2015 to 2016.

Percentage of Antimicrobial Resistance and Statistical Comparison of Resistant Isolates (Z-Test for Proportions)								
	Swine			Human			Z-Score	*p*-Value
Antimicrobial (Family) ^1^	Pathogenic n = 62 (6.31%)	Commensal n = 921 (93.69%)	Total N = 983 (100%)	Pathogenic n = 95 (22.35%)	Commensal n = 330 (77.65%)	Total N = 425 (100%)		
Ampicillin [B]	4.47 (44)	50.97 (501)	55.44 (545)	13.88 (59)	44.0 (187)	57.88 (246)	−0.80	0.42
Ceftazidime [B]	0.00 (0)	2.54 (25)	2.54 (25)	3.77 (16)	10.82 (46)	14.59 (62)	−8.74	<0.0001
Cefotaxime [B]	0.00 (0)	2.44 (24)	2.44 (24)	2.82 (12)	8.71 (37)	11.53 (49)	−7.19	<0.0001
Gentamicin [A]	1.42 (14)	36.42 (358)	37.84 (372)	9.88 (42)	36.47 (155)	46.35 (197)	−2.94	0.0032
Ciprofloxacin [Q]	2.95 (29)	20.44 (201)	23.39 (230)	1.65 (7)	13.65 (58)	15.30 (65)	3.39	0.0007
Nalidixic acid [Q]	5.49 (54)	73.45 (722)	78.94 (776)	14.12 (60)	51.29 (218)	65.41 (278)	5.42	<0.0001
Cotrimoxazole [S] ^2^	3.96 (39)	49.95 (491)	53.91 (530)	12.94 (55)	40.94 (174)	53.88 (229)	0.058	0.95
Tetracycline [T]	5.19 (51)	66.73 (656)	71.92 (707)	14.59 (62)	57.18 (243)	71.77 (305)	0.021	0.98
MDR ^3^	4.78 (47)	61.44 (604)	66.22 (651)	14.82 (63)	49.88 (212)	64.71 (275)	0.51	0.61

^1^ Abbreviations: A, aminoglycosides; B, β-lactams; Q, quinolones; S, sulfonamides; T, tetracyclines. ^2^ Cotrimoxazole = Trimethoprim-sulfamethoxazole. ^3^ MDR = multidrug resistance.

**Table 4:** Multidrug resistance of *E. coli* isolates from swine colonization and human diarrhea in Jiutepec, Morelos, Mexico, from 2015 to 2016. The antimicrobial families defined by the Clinical and Laboratory Standards Institute (CLSI) are used.

Group (%)	Number of Antibiotic Resistances	No. of Isolates (%)	Most Frequent Pattern of Antimicrobial Resistance (No. of Isolates) ^1^
Swine n = 651 (66.22)	3	251 (25.54)	NAL-SXT-TET (48) GEN-NAL-TET (43)
	4	187 (19.02)	AMP-NAL-SXT-TET (79)
	5	129 (13.12)	AMP-CIP-NAL-SXT-TET (63)
	6	70 (7.12)	AMP-GEN-CIP-NAL-SXT-TET (65)
	7	8 (0.81)	AMP-CAZ-CTX-CIP-NAL-SXT-TET (4)
	8	6 (0.61)	AMP-CAZ-CTX-GEN-CIP-NAL-SXT-TET (6)
Humans n = 275 (64.71)	3	95 (22.35)	AMP-NAL-TET (19)
			GEN-NAL-TET (19)
	4	83 (19.53)	AMP-NAL-SXT-TET (31)
	5	43 (10.12)	AMP-GEN-NAL-SXT-TET (23)
	6	22 (5.18)	AMP-GEN-CIP-NAL-SXT-TET (9)
	7	14 (3.29)	AMP-CAZ-CTX-CIP-NAL-SXT-TET (6)
	8	18 (4.24)	AMP-CAZ-CTX-GEN-CIP-NAL-SXT-TET (18)

^1^ Abbreviations: AMP, ampicillin; CAZ, ceftazidime; CTX, cefotaxime; GEN, gentamicin; CIP, ciprofloxacin; NAL, nalidixic acid; SXT, cotrimoxazole; TET, tetracycline.

**Table 5:** Frequency of virulence genes among isolates of multidrug-resistant diarrheagenic *E. coli* from swine and human diarrhea in Jiutepec, Morelos, Mexico, from 2015 to 2016.

Pathotypes ^1^	Virulence Genes	Swine N = 983			Human N = 425		
		Total Diarrheagenic *E. coli* n = 62 (6.31%) ^2^			Total Diarrheagenic *E. coli* n = 95 (22.35%) ^2^		
		MDR n = 47 (75.8%) ^3^	No MDR n = 15 (24.19%) ^3^	Total	MDR n = 63 (66.31%) ^3^	No MDR n = 32 (33.68%) ^3^	Total
EPEC	*eae*	34 (54.84%)	10 (16.13%)	44 (70.97%)	11 (11.58%)	6 (6.32%)	17 (17.90%)
EHEC	*sxt1*	4 (6.45%)	3 (4.84%)	7 (11.29%)	2 (2.10%)	3 (3.16%)	5 (5.26%)
	*sxt2*	6 (9.68%)	0 (0.00%)	6 (9.68%)	2 (2.10%)	0 (0.00%)	2 (2.10%)
	*stx1/stx2*	1 (1.61%)	0 (0.00%)	1 (1.61%)	0 (0.00%)	0 (0.00%)	0 (0.00%)
ETEC	*elt*	1 (1.61%)	1 (1.61%)	2 (3.22%)	6 (6.32%)	4 (4.21%)	10 (10.53%)
	*est*	0 (0.00%)	0 (0.00%)	0 (0.00%)	0 (0.00%)	1 (1.05%)	1 (1.05%)
EIEC	*ipa*H	1 (1.61%)	1 (1.61%)	2 (3.22%)	7 (7.37%)	5 (5.26%)	12 (12.63%)
EAEC	*aat*	0 (0.00%)	0 (0.00%)	0 (0.00%)	8 (8.43%)	2 (2.10%)	10 (10.53%)
DAEC	*daa*	0 (0.00%)	0 (0.00%)	0 (0.00%)	27 (28.42%)	11 (11.58%)	38 (40.00%)

^1^ Abbreviations: EPEC, enteropathogenic *E. coli*; EHEC, enterohemorrhagic *E. coli*; ETEC, enterotoxigenic *E. coli*; EIEC, enteroinvasive *E. coli*; EAEC, enteroaggregative *E. coli*; DAEC, diffusely adhering *E. coli*. ^2^ Frequency is calculated by dividing the numbers (n) by the total number of *E. coli* strains identified in sampling (N = 983, swine; N = 425, human). ^3^ Frequency is calculated by dividing the numbers (n) by the total number of diarrheagenic *E. coli* strains identified in sampling (n = 62, swine; n = 95, human).

## Data Availability

The original contributions presented in this study are included in the article. Further inquiries can be directed to the corresponding author.
